# β-GPA: AN AMPK ACTIVATOR WITH POTENTIAL EFFECTS ON HEALTHSPAN AND FUNCTION

**DOI:** 10.1093/geroni/igz038.337

**Published:** 2019-11-08

**Authors:** Jonathan Dorigatti, Kevin Thyne, Yuhong Liu, Adam B Salmon

**Affiliations:** 1 University of Texas, Health Science Center at San Antonio, San Antonio, Texas, United States; 2 University of Texas Health at San Antonio, San Antonio, Texas, United States

## Abstract

β-Guanidinopropionic acid (β-GPA) is a naturally occurring compound reported to activate AMP Activated Protein Kinase (AMPK) signaling in vivo. Acute administration of β-GPA in young animals has been reported to improve multiple functional measures including a switch to oxidative fatigue-resistant muscle fibers, improved glucose uptake, and increased mitochondrial biogenesis. However, it is unknown if β-GPA may promote healthy aging or prevent late-life functional decline. To address this knowledge gap, we tested the effects of β-GPA on mitochondrial energetics and cellular function in young and old genetically heterogenous mice (HET3). Both age groups were fed either 1% β-GPA or control chow for ~5 months and basic functional parameters including metabolism were assessed. β-GPA treatment decreased lean and fat mass in young males, but prevented late-life losses in these parameters in old animals. Notably, glycated hemoglobin (HbA1c) levels were lower in treated young and old males suggesting improved glucose homeostasis. Citrate synthase activity was also higher in old males fed β-GPA suggesting increased mitochondrial biogenesis. At the molecular level, mitochondrial Complex I expression decreased with β-GPA treatment in old males versus controls. High resolution respirometry revealed generally decreased respiration in old animals compared to young and decreased Complex 1 coupled respiration in soleus of β-GPA treated young but not old males. These findings indicate that the mitochondrial effects attributed to β-GPA may be mediated by its action on Complex I. While treatment outcomes varied in young and old males these results suggest β-GPA may prove beneficial in combating age-related declines in function.

